# Identification of Genetic Suppressors of the *Sin3A* Knockdown Wing Phenotype

**DOI:** 10.1371/journal.pone.0049563

**Published:** 2012-11-15

**Authors:** Aishwarya Swaminathan, Valerie L. Barnes, Stephanie Fox, Sarah Gammouh, Lori A. Pile

**Affiliations:** 1 Department of Microbiology, University of Massachusetts, Amherst, Massachusetts, United States of America; 2 Department of Biological Sciences, Wayne State University, Detroit, Michigan, United States of America; University of Sheffield - MRC Centre for Developmental and Biomedical Genetics, United Kingdom

## Abstract

The role of the Sin3A transcriptional corepressor in regulating the cell cycle is established in various metazoans. Little is known, however, about the signaling pathways that trigger or are triggered by Sin3A function. To discover genes that work in similar or opposing pathways to *Sin3A* during development, we have performed an unbiased screen of deficiencies of the *Drosophila* third chromosome. Additionally, we have performed a targeted loss of function screen to identify cell cycle genes that genetically interact with *Sin3A*. We have identified genes that encode proteins involved in regulation of gene expression, signaling pathways and cell cycle that can suppress the curved wing phenotype caused by the knockdown of *Sin3A*. These data indicate that Sin3A function is quite diverse and impacts a wide variety of cellular processes.

## Introduction

Histone acetylation levels are maintained by the opposing activities of histone lysine acetyltransferases (KATs) and histone deacetylases (HDACs). Modulation of acetylation levels that affect regulation of gene expression has been shown to be an important process during *Drosophila* development. Rearing *Drosophila* larvae on varying concentrations of the HDAC inhibitor Trichostatin A (TSA) results in death at high concentrations or in delayed development and a notched wing phenotype in adults at low TSA concentrations, suggesting that the deacetylase activity of HDAC complexes is important for regulating viability and developmental events [1]. *Sin3A*, which encodes a corepressor component of HDAC complexes, is an essential gene for embryo and larvagenesis [2], [3], [4]. The Sin3A complex is hypothesized to regulate developmental processes via its association with the HDAC Rpd3.

**Figure 1:**
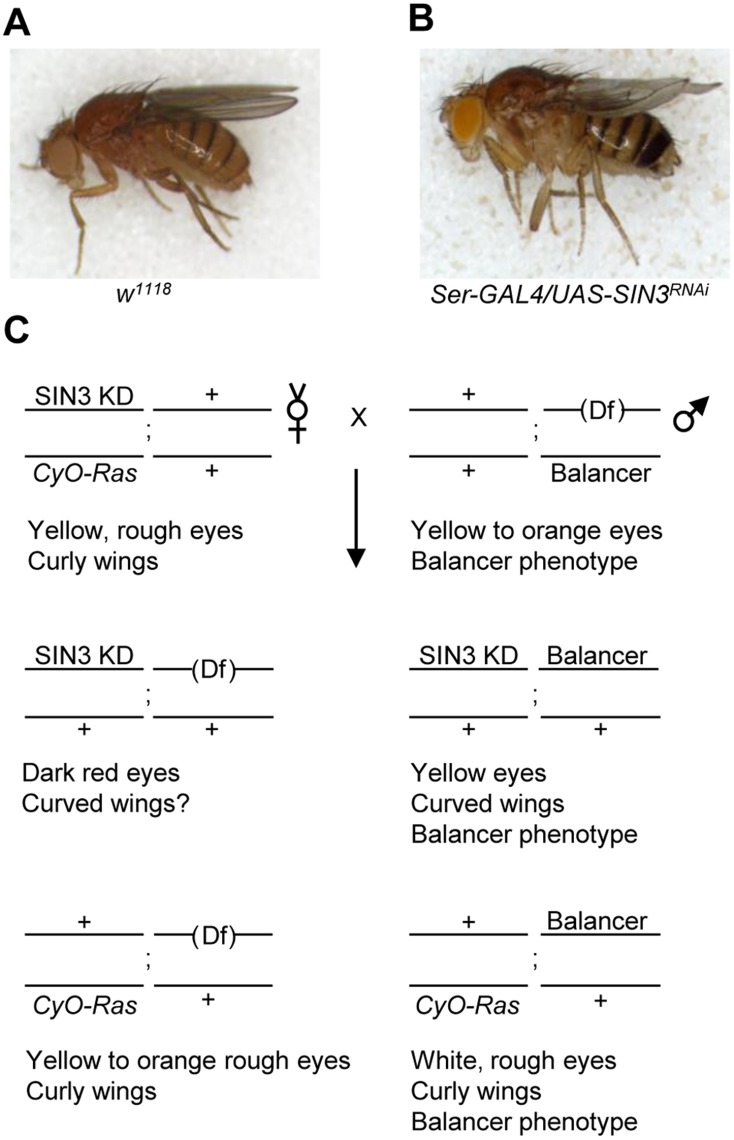
General scheme of the screen. (A, B) Images of wild type (*w^1118^*) straight wing and SIN3 KD (*Ser-GAL4* X *UAS-SIN3^RNAi^*) curved wing *Drosophila* as indicated. (C) SIN3 KD/*CyO-Ras* females were crossed to males heterozygous for either a deletion that removed multiple genes (phase I and II) or either a loss of function allele or RNAi line of a single gene (phase III) balanced over a third chromosome balancer that was *TM3-Sb*, *TM3-Ser*, *TM2-Ubx* or *TM6-Tb*. The resulting progeny that are *Sin3A* knockdown and carry a third chromosome deletion were scored for the curved wing phenotype. In phase I of the screen, each deletion on the third chromosome was associated with a gene resulting in yellow to orange eye color. In the case of phase II, all the deletions tested were in a *w* background. The majority of RNAi lines were homozygous and the cross yielded progeny of two genotypes.

Sin3A has been implicated in the regulation of signaling that directs developmental pathways. *Drosophila Sin3A* was first isolated in a screen to identify components of MAP kinase signaling during eye development [2]. Loss of *Sin3A* enhances the rough eye phenotype caused by a mutation in *sina*, a gene required for photoreceptor specification directed by MAP kinase signaling. Sin3A has also been implicated in the regulation of development via steroid hormone signaling. SMRTER, a corepressor of genes induced by the hormone ecdysone, brings about transcriptional repression by recruiting the Sin3A complex to target genes [5]. Sin3A colocalizes with SMRTER on polytene chromosomes [6]. The recruitment of Sin3A to ecdysone responsive genes is reduced upon activation by the steroid hormone. Sin3A levels are restored at these genes when these are repressed. *Sin3A* has also been a positive hit in a number of genome-wide RNA interference (RNAi) screens looking for genes encoding factors involved in a number of distinct developmental and signaling pathways. RNAi screens in *Drosophila* embryos have implicated *Sin3A* in neural and cardiac development [7], [8], [9]. *Sin3A* was identified in an adult *Drosophila* screen looking for factors in Notch signaling [10]. A putative role of Sin3A in ERK and JNK signaling comes from screens in cultured cells [11], [12]. Taken together, results from multiple researchers highlight a critical role of Sin3A in *Drosophila* development.

**Table 1:** Genes involved in multiple cellular processes genetically interact with *Sin3A*.

Function^a^	CG Number^b^	Gene Symbol
Transcription	CG12809	*nerfin-2*
	CG2702	*Pbp95*
	CG10488	*eyg*
	CG10704	*toe*
	CG10390	*mia*
	CG43662	*Rpb4*
	CG9461	*FBX011*
	CG7467	*osa*
	CG4107	*Pcaf*
	CG3909	*CG3909*
	CG5358	*Art4*
	CG1070	*Alh*
	CG11033	*Kdm2*
Signaling	CG5974	*pII*
	CG11848	*Bili*
	CG31110	*5PtaseI*
	CG12876	*ALiX*
	CG7910	*CG7910*
Cell Proliferation and Division	CG2669	*hd*
	CG10061	*Sas-4*
	CG6875	*asp*
	CG5814	*CycB3*
GTPase activity	CG1081	*Rheb*
	CG1250	*sec23*
Apoptosis	CG10233	*rtp*
DNA repair	CG10018	*Snm1*
Translation	CG2957	*mRpS9*
Larval Development	CG2723	*ImpE3*
Metabolism	CG5804	*CG5804*
	CG1152	*Gld*
	CG1939	*Dpck*
Proteolysis	CG11951	*CG11951*
Chitin binding	CG32024	*CG32024*
Unknown	CG32023	*CG32023*
	CG10053	*CG10053*
	CG11993	*Mst85C*
	CG12347	*CG12347*
	CG14463	*CG14463*

a Functional process information was obtained from the listing for the individual gene on FlyBase [20].

b Flies carrying mutations or having reduced expression of these genes suppressed the *Sin3A* knockdown curved wing phenotype but did not have a wing phenotype in wild type background. For percent suppression, refer to Table S1.

Sin3A also plays an important role in regulating the cell cycle. In *Drosophila*, *Sin3A* knockdown by RNAi results in a G2 arrest in S2 cells [13]. Ubiquitous knockdown of *Sin3A* results in embryonic lethality, presumably due to defects in cell proliferation [4]. In wing discs, loss of *Sin3A* results in a decrease in the number of mitotic cells leading to fewer cells in the adult wing [14]. This wing phenotype is partially suppressed by the overexpression of G2 phase regulators including *String* (*stg*) and *cdc2*. These results reinforce the role of Sin3A in regulating the G2 phase, but also suggest that Sin3A may regulate other phases of the cell cycle. The mechanism by which this potential regulation is brought about is unknown.

Although Sin3A and histone acetylation have been implicated in various developmental processes, the specific pathways regulated by the Sin3A HDAC complex during development are unknown. Genetic screens in *Drosophila* have served as a valuable tool in identifying novel gene function. Functions of Sin3A and components of the Sin3A complex have been identified in a variety of screens and have helped shed light on their potential roles in *Drosophila* development [2], [3], [15], [16], [17], [18]. To identify novel pathways in which Sin3A may function and to find novel genes that interact with *Sin3A*, we performed an unbiased screen of the third chromosome. We identified several genes encoding factors that have been shown to play a role in various processes including regulation of transcription, regulation of signaling and cell division. We have also performed a targeted screen to further analyze the role of Sin3A in regulating the cell cycle in the developing wing disc. Results of the targeted screen indicate that Sin3A plays a role in regulating multiple phases of the cell cycle. These data provide insight into the role of Sin3A, as well as identify novel genes important in wing development.

**Figure 2:**
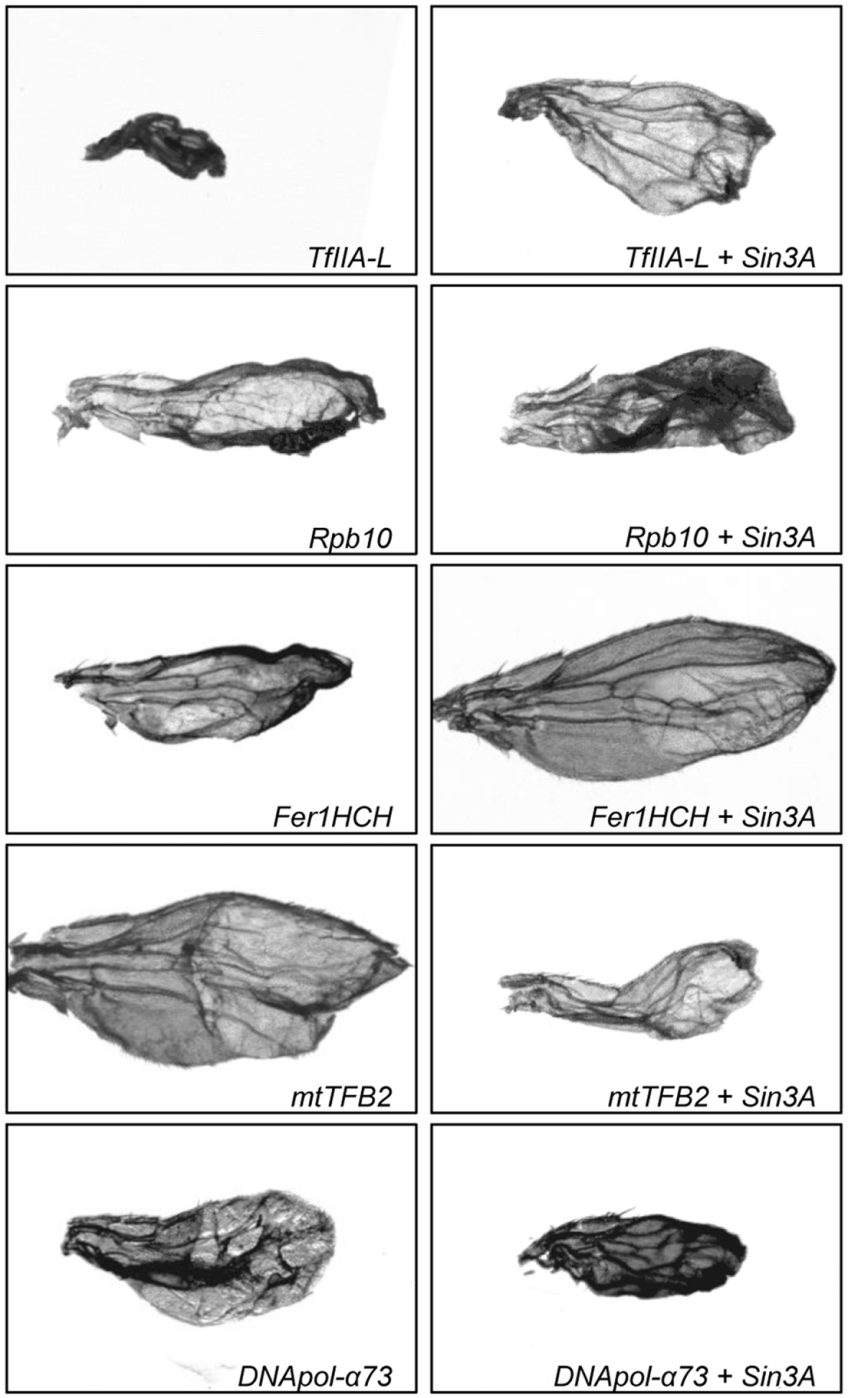
Some genes along the third chromosome are required for normal wing development and interact with *Sin3A*. Images of representative wings from progeny of *Ser-GAL4* X *UAS-RNAi* (left panels) and from SIN3 KD X *UAS-RNAi* (right panels) of the indicated gene.

**Table 2:** Genes involved in negative regulation of the Wnt pathway genetically interact with *Sin3A*.

CG Number	Gene Symbol	SIN3 KD I^a^		SIN3 KD II^a^	
		RNAi	LOF	RNAi	LOF
CG11848	*Bili*	55±6^b^	28±11^b^	55±10^b^	35±5^b^
			24±9		18±3
CG6193	*Apc2*	30±12	n.t.	17±9	n.t.
CG1451	*Apc*	n.t.	4±1	n.t.	6±3
CG7926	*Axn*	11±6	n.t.	3±1	n.t.
CG3352	*ft*	0^c^	n.t.	0^c^	n.t.
CG8384	*gro*	25±19	n.t.	40±3	n.t.
CG34403	*pan*	48±6	n.t.	52±17	n.t.
CG10225	*RanBP3*	53±8	n.t.	45±6	n.t.
CG2621	*sgg*	0^c^	7±2	0^c^	8±2
CG11895	*stan*	7±8	n.t.	10±1	n.t.
CG13345	*tum*	0^c^	n.t.	0^c^	n.t.

a SIN3 KD I and II/*CyO-Ras* females were crossed to males carrying an RNAi or loss of function (LOF) allele for the indicated gene.

b The percentage of straight winged flies in the progeny of the cross that are knocked down for *Sin3A* and for the indicated gene is given. Results are an average of three trials. n>100. Standard deviation is indicated.

c Flies had a wing phenotype that was neither straight nor curved.

n.t., not tested.

## Materials and Methods

### Drosophila Stocks

*Drosophila melanogaster* stocks were maintained and crosses were performed according to standard laboratory procedures. The following stocks were used: *UAS-SIN3^RNAi-I^* [4], *UAS-SIN3^RNAi-II^*, SIN3 KD I [14] and SIN3 KD II (construction described below), from the Bloomington stock center: *w^1118^*, *Ser-GAL4* (#6791), Isogenic/DrosoDel deficiency kit stocks which includes those designated with “ED” [19] Df(3R)ED2 (#6962), Df(3R)ED5577 (#8029), Df(3L)ED4079 (#8046), Df(3L)ED201 (#8047), Df(3L)ED4177 (#8048), Df(3L)ED4191 (#8049), Df(3L)ED4196 (#8050), Df(3L)ED202 (#8051), Df(3L)ED4238 (#8052), Df(3L)ED207 (#8053), Df(3L)ED4256 (#8054), Df(3L)ED4284 (#8056), Df(3L)ED4288 (#8057), Df(3L)ED4293 (#8058), Df(3L)ED208 (#8059), Df(3L)ED4341 (#8060), Df(3L)ED210 (#8061), Df(3L)ED4342 (#8062), Df(3L)ED211 (#8063), Df(3L)ED4408 (#8065), Df(3L)ED4421 (#8066), Df(3L)ED4470 (#8068), Df(3L)ED4475 (#8069), Df(3L)ED4483 (#8070), Df(3L)ED215 (#8071), Df(3L)ED4486 (#8072), Df(3L)ED4543 (#8073), Df(3L)ED217 (#8074), Df(3L)ED21 (#8075), Df(3L)ED220 (#8077), Df(3L)ED4606 (#8078), Df(3L)ED223 (#8079), Df(3L)ED224 (#8080), Df(3L)ED225 (#8081), Df(3L)ED4782 (#8082), Df(3L)ED4786 (#8083), Df(3L)ED4789 (#8084), Df(3L)ED4799 (#8085), Df(3L)ED228 (#8086), Df(3L)ED229 (#8087), Df(3L)ED4858 (#8088), Df(3L)ED230 (#8089), Df(3L)ED231(#8090), Df(3R)ED5092 (#8091), Df(3R)ED5066 (#8092), Df(3R)ED5095 (#8093), Df(3R)ED5558 (#8095), Df(3L)ED4287 (#8096), Df(3L)ED4502 (#8097), Df(3L)ED4674 (#8098), Df(3L)ED4685 (#8099), Df(3L)ED4710 (#8100), Df(3L)ED4978 (#8101), Df(3L)ED5017 (#8102), Df(3R)ED5177 (#8103), Df(3R)ED5780 (#8104), Df(3R)ED6232 (#8105), Df(3R)ED6242 (#8107), Df(3R)ED5138 (#8680), Df(3R)ED5196 (#8681), Df(3R)ED5230 (#8682), Df(3R)ED5911 (#8683), Df(3R)ED6096 (#8684), Df(3R)ED7665 (#8685), Df(3R)ED5429 (#8919), Df(3R)ED5559 (#8920), Df(3R)ED5623 (#8921), Df(3R)ED5942 (#8922), Df(3R)ED6085 (#8923), Df(3R)ED6093 (#8924), Df(3R)ED6316 (#8925), Df(3R)ED5514 (#8957), Df(3R)ED5622 (#8959), Df(3R)ED6265 (#8960), Df(3R)ED6310 (#8961), Df(3R)ED6076 (#8962), Df(3R)ED6103 (#8963), Df(3R)ED6025 (#8964), Df(3R)ED5156 (#8965), Df(3R)ED5147 (#8967), Df(3R)ED5516 (#8968), Df(3L)ED4413 (#9070), Df(3L)ED4515 (#9071), Df(3L)ED4528 (#9072), Df(3L)ED4529 (#9073), Df(3L)ED4534 (#9074), Df(3R)ED5020 (#9075), Df(3R)ED5223 (#9076), Df(3R)ED5330 (#9077), Df(3R)ED5438 (#9078), Df(3R)ED5454 (#9080), Df(3R)ED5472 (#9081), Df(3R)ED5474 (#9082), Df(3R)ED5506 (#9083), Df(3R)ED5518 (#9084), Df(3R)ED5554 (#9085), Df(3R)ED5591 (#9086), Df(3R)ED5610 (#9087), Df(3R)ED5612 (#9088), Df(3R)ED5613 (#9089), Df(3R)ED5644 (#9090), Df(3R)ED6090 (#9091), Df(3R)ED6091 (#9092), Df(3R)ED5705 (#9152), Df(3R)ED1025 (#9159), Df(3L)ED4415 (#9194), Df(3R)ED5021 (#9196), Df(3R)ED5046 (#9197), Df(3R)ED5142 (#9198), Df(3R)ED5187 (#9199), Df(3R)ED5220 (#9200), Df(3R)ED5221 (#9201), Df(3R)ED5327 (#9202), Df(3R)ED5331 (#9203), Df(3R)ED5339 (#9204), Df(3R)ED5573 (#9206), Df(3R)ED5785 (#9207), Df(3R)ED5815 (#9208), Df(3R)ED6255 (#9210), Df(3R)ED6220 (#9211), Df(3L)ED4536 (#9214), Df(3R)ED5495 (#9215), Df(3R)ED5071 (#9224) Df(3R)ED5301 (#9225), Df(3R)ED5100 (#9226), Df(3R)ED5428 (#9227), Df(3R)ED5634 (#9228), Df(3R)ED5642 (#9279), Df(3R)ED6237 (#9280), Df(3R)ED5296 (#9338), Df(3R)ED5197 (#9339), Df(3R)ED6187 (#9347), Df(3L)ED4457 (#9355), Df(3R)ED6235 (#9478), Df(3R)ED6027 (#9479), Df(3R)ED6052 (#9480), Df(3R)ED10639 (#9481), Df(3R)ED10642 (#9482), Df(3R)ED10838 (#9485), Df(3R)ED10845 (#9487), Df(3R)ED10555 (#23714), Df(3R)ED5416 (#24136), Df(3R)ED10566 (#24138), Df(3R)ED5938 (#24139), Df(3R)ED6058 (#24140), Df(3R)ED6332 (#24141), Df(3R)ED6346 (#24142), Df(3R)ED6361 (#24143), Df(3R)Exel6146 (#7625), Df(3R)Exel6154 (#7633), Df(3R)Exel6155 (#7634), Df(3R)Exel6200 (#7679), Df(3R)Exel6201 (#7680), Df(3R)Exel6205 (#7684), Df(3R)Exel6206 (#7685), Df(3R)Exel6208 (#7686), Df(3R)Exel6212 (#7690), Df(3R)Exel6263 (#7730), Df(3R)Exel9029 (#7981), Df(3L)BSC130 (#9295), Df(3R)BSC196 (#9622), Df(3R)BSC177 (#9692), Df(3R)BSC221 (#9698), Df(3R)BSC222 (#9699), Df(3R)BSC179 (#23146), Df(3R)BSC176 (#24334), Df(3R)BSC318 (#24344), Df(3R)BSC397 (#24421), Df(3R)BSC465 (#24969), Df(3R)BSC466 (#24970), Df(3R)BSC493 (#24997), Df(3R)BSC513 (#25017), Df(3R)BSC548 (#25076), Df(3L)BSC612 (#25687), Df(3R)BSC633 (#25724), Df(3R)BSC650 (#25740), Df(3R)BSC686 (#26538), Df(3R)BSC729 (#26581), *UAS-GFP^RNAi^* (#9331), *elm^EY07304^* (#19861), *sec23^EY06757^* (#19921), *Gld^n2^* (#2439), *pll^7^* (#3112), *spz^2^* (#3115), *ash^2^* (#4584), *ash2^EY03971^* (#15697), *CycB3^2^* (#6635), *Rheb^AV4^* (#9690), *Sas-4^s2214^* (#12119), *mia^EY07883^* (#16865), *CG11951^f00339^* (#18316), *Bili^MB01370^* (#23079), *Bili^MB07242^* (#25639), *CG7910^MB06548^* (#25514), *neur^11^* (#2747), *kto^1^* (#3618), *skd^2^* (#5047), *CycB^2^* (#6630), *Ets98B^MB04306^* (#24806), *TfIIA-L^MB07587^* (#25559), *Pcaf^Q186st^* (#9334), *Pcaf^C137T^* (#9335), *ru^1^* (#575), *rho^ve−1^* (#628), *toe^MB03498^* (#24002), *eyg^1^* (#503), *Kat-60^UY1645^* (#7345), *Ref1^02267^* (#11562), *Dpck^e01273^* (#17934), *Alh^r13^* (#2418), *Sas^15^* (#2098), *CG10055^EY19442^* (#22158), *mRpS9^EY02265^* (#15861), *CG14463^G20251^* (#31865), *asp^1^* (#1972), *TRiP.JF03169* (*UAS-asp^RNAi^,* #28741), *Ada2b^3412^* (#17125), *CG9603^e03209^* (#18133), *mRpL19^03^* (#23280), *Kdm2^KG04325^* (#13589), *Kdm2^EY01336^* (#15510), *osa^2^* (#3616), *CG7379^KG03341^* (#14430), *MED17^s2956^* (#10307), *MED20^C6R20^* (#4967), *Mdh2^EY01940^* (#15383), *Rpb4^MB03453^* (#24068), *ALiX^EY10362^* (#17675), *Ets98B^MB04306^* (#24806), *Brd8^G19099^* (#31838), *cdc2c^2^* (#6632), *cdc16^EY12544^* (#20753), *cdc16^MB09129^* (#26144) *Apc^MB08754^* (#264000), *sgg^MB03827^* (#24662), *TRiP.HMS01095* (*UAS-CycC^RNAi^*, #33753), *MED10^G18634^* (#27446), *MED1^EY20943^* (#22437), *MED23^KG00948^* (#13302), *MED24^BG01670^* (#12847). From the Vienna *Drosophila* Research Center: *UAS-CG5804^RNAi^* (#23587GD), *UAS-CG32023^RNAi^* (#108338KK), *UAS-CG32024^RNAi^* (#102205KK), *UAS-hd^RNAi^* (#47309GD), *UAS-kkv^RNAi^* (#100327KK), *UAS-RpII18^RNAi^* (#105937KK), *UAS-Mms19^RNAi^* (#108131KK), *UAS-rtp^RNAi^* (#109000KK), *UAS-Snm1^RNAi^* (#37591GD), *UAS-Mia^RNAi^* (#100313KK), *UAS-ash2^RNAi^* (#7141GD, 100718KK), *UAS-Rpb10^RNAi^* (#102010KK), *UAS-polybromo^RNAi^* (#108618KK), *UAS-Bili^RNAi^* (#101424KK), *UAS-danr^RNAi^* (#11515GD), *UAS-Ser^RNAi^* (#108348KK), *UAS-DNApol-α73^RNAi^* (#108579KK), *UAS-TfIIA-L^RNAi^* (#108355KK), *UAS-woc^RNAi^* (#20995GD), *UAS-Art4^RNAi^* (#107009KK), *UAS-CG3909^RNAi^* (#104387KK), *UAS-FBXO11^RNAi^* (#24039GD), *UAS-CG9467^RNAi^* (#45807GD), *UAS-Mical^RNAi^* (#105837KK), *UAS-mtTFB2^RNAi^* (#107086KK), *UAS-nerfin-2^RNAi^* (#101434KK), *UAS-Pbp95^RNAi^* (#33558GD), *UAS-ImpE3^RNAi^* (#16403GD), *UAS-Fer1^RNAi^* (#104963KK), *UAS-Sas-4^RNAi^* (#106051KK), *UAS-CG18012^RNAi^* (#20580GD), *UAS-Rim^RNAi^* (#39385GD), *UAS-CG17801^RNAi^* (#29501GD), *UAS-CG12347^RNAi^* (#106206KK), *UAS-CG7357^RNAi^* (#100097KK), *UAS-hdc^RNAi^* (#104322KK), *UAS*-*Fer1HCH^RNAi^* (#102406KK), *UAS-Mst85C^RNAi^* (#6493GD), *UAS-neur^RNAi^* (#108239KK), *UAS-Kdm2^RNAi^* (#109295KK), *UAS-cycB3^RNAi^* (#108009KK), *UAS-Brd8^RNAi^* (#104879KK), *UAS-Apc2^RNAi^* (#100104KK), *UAS-Axn^RNAi^* (#77486GD), *UAS-RanBP3^RNAi^* (#38363GD), *UASUAS-ft^RNAi^* (#108863KK), *UAS-gro^RNAi^* (#6316GD), *UAS-pan^RNAi^* (#108679KK), *UAS-sgg^RNAi^* (#101538KK), *UAS-stan^RNAi^* (#107993KK), *UAS-tum^RNAi^* (#106850KK) *UAS-Pcaf^RNAi^* (#108943KK), *UAS-Prosβ7^RNAi^* (#101990KK), *UAS-sas^RNAi^* (#100901KK), *UAS-CG10053^RNAi^* (#17972GD) *UAS-rn^RNAi^* (#109848), *UAS-CG10903^RNAi^* (#109610), *UAS-Ast^RNAi^* (#103215KK), *UAS-5PtaseI^RNAi^* (#100802KK), *UAS-Dp^RNAi^* (#12722GD), *UAS-CycE^RNAi^* (#52662GD), *UAS-Rbf^RNAi^* (#10696GD), *UAS-cdc2c^RNAi^* (#104959KK), *UAS-CycB^RNAi^* (#43772GD), *UAS-CycA^RNAi^* (#32421GD), *UAS-cdc16^RNAi^* (#103583KK), *UAS-CycJ^RNAi^* (#31216GD), *UAS-Cdk8^RNAi^* (#107187KK), *UAS-kto^RNAi^* (#23143GD).

### Generation of SIN3 KD I and SIN3 KD II Flies

Generation of constitutive wing imaginal disc *Sin3A* knockdown (SIN3 KD) recombinant flies is described in [14]. These flies are referred to as SIN3 KD I in this current study. SIN3 KD II recombinants were generated in a similar fashion by crossing *Ser-GAL4*/*UAS-SIN3^RNAi-II^* females to *CyO-Ras/Sco* males. Recombinant progeny were scored on the basis of eye color. Potential recombinants were verified by crossing to *w^1118^* and monitoring the penetrance of the curved wing phenotype in the progeny.

**Figure 3:**
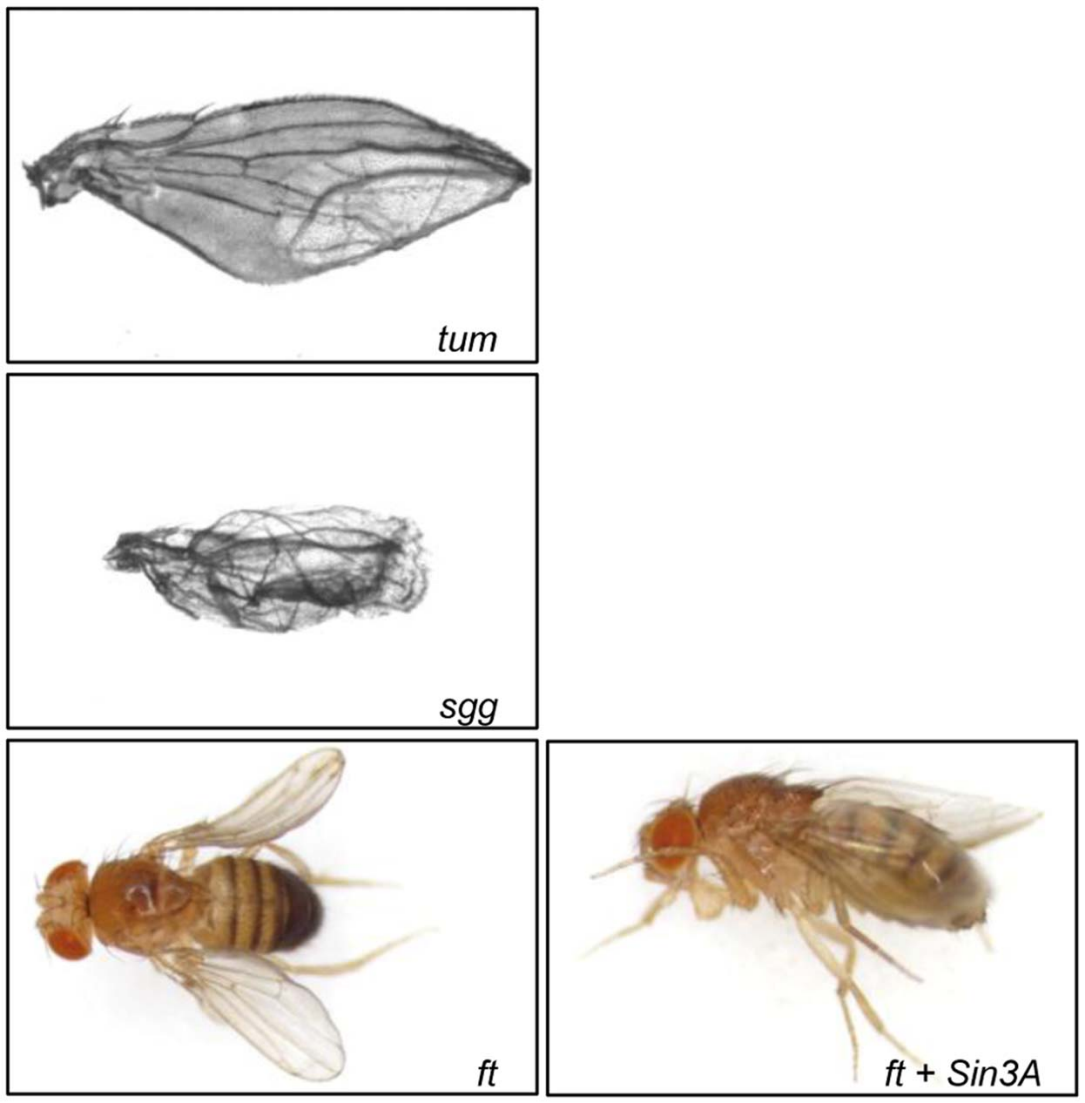
Reduced expression of negative regulators of the Wnt signaling pathway results in abnormal wing morphology. Images of representative wings from progeny of *Ser-GAL4* X *UAS-RNAi* (left panels) and from SIN3 KD X *UAS-RNAi* (right panel) of the indicated gene. For *tum and sgg*, the wing phenotype of the double knockdown was the same as for the single gene knockdown. The phenotype of the *ft, Sin3A* double knockdown is similar to the SIN3 KD curved wing as shown.

**Figure 4:**
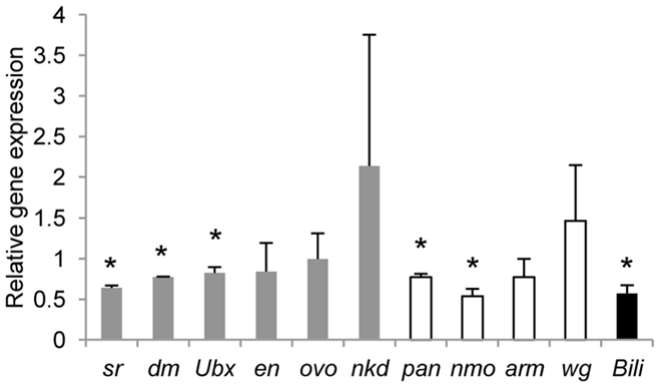
Loss of *Sin3A* results in down regulation of genes involved in the Wnt pathway. qRT-PCR analysis of the mRNAs of the indicated genes. mRNA from control *w^1118^* and *Sin3A* knockdown wing discs was reverse transcribed into cDNA to use as template in the PCR. Gene expression in *Sin3A* knockdown wing discs relative to *w^1118^* is indicated. Expression was normalized to *Taf1* and *Pgk* expression. n = 3. Error bars indicate standard deviation. (*) 0.006<p<0.02. Gray bars, Wnt targets. White bars, Wnt targets and effectors. Black bar, Wnt effector.

### Imaging

Wing images (63X) and adult fly images (30X) were taken with an Olympus DP72 camera coupled to an Olympus SZX16 microscope.

### Reverse Transcription PCR Assay

Total RNA was extracted from wing discs isolated from wandering third instar larvae using the RNeasy mini kit (Qiagen). cDNA was generated from total RNA using the ImProm-II Reverse Transcription System (Promega) with random hexamers. The cDNA was used as template in a quantitative real-time PCR (qPCR) assay. The analysis was performed using ABsolute SYBR Green ROX master mix (Fisher Scientific) and carried out in a Stratagene Mx3005P real-time thermocycler. Primers used for analysis are given in Table S1. *Taf1* was used to normalize cDNA amounts in the comparative analysis.

**Table 3:** *Sin3A* interacts genetically with cell cycle regulators.

CG Number	Gene Symbol	Cell Cycle Phase	SIN3 KD I^a^		SIN3 KD II^a^	
			RNAi	LOF	RNAi	LOF
CG4654	*Dp*	G1/S [113]	0^b^	n.t.	0^b^	n.t.
CG7405	*CycE*	G1/S [114]	35±9	n.t.	38±4	n.t.
CG7413	*Rbf*	G1/S [115]	0	n.t.	0	n.t.
CG10498	*cdc2c*	G1/S [116]	6^c^	2±1	0^c^	3±1
CG3510	*CycB*	G2/M [117], [118]	11±1	n.t.	15±3	n.t.
CG5940	*CycA*	G2/M [119]	0c,d	n.t.	0c,d	n.t.
CG6759	*cdc16*	M [84]	0^c^	26±1	0^c^	28±1
				20±1		23±3
CG10308	*CycJ*	M [120]	23±1	n.t.	44±9	n.t.
CG5814	*CycB3*	M [80], [81]	11±4	2±2	13±2	8±3

a SIN3 KD I and II/*CyO-Ras* females were crossed to males carrying an RNAi or loss of function (LOF) allele for the indicated cell cycle regulator.

b The percentage of straight winged flies in the progeny of the cross that are knocked down for *Sin3A* and for the indicated gene is given. Results are an average of three trials. n>100. Standard deviation is indicated.

c Flies had a wing phenotype that was neither straight nor curved.

d The double knockdown resulted in a partial lethal phenotype.

n.t., not tested.

## Results and Discussion

Reduction of Sin3A protein levels by RNAi knockdown in cells of the wing imaginal disc results in a curved wing phenotype in the adult fly (Figure 1) [14]. This mild but completely penetrant phenotype can be modified by mutations in or overexpression of genes that interact with *Sin3A*. As the first step to identify novel genes that genetically interact with *Sin3A*, we performed an unbiased screen of the third chromosome using the isogenic deficiency kit [19]. Our initial goal was to determine genomic regions which when deleted modify the *Sin3A* knockdown curved wing phenotype. For the screen we utilized flies that had constitutive knockdown of *Sin3A* in wing imaginal disc cells, referred to as SIN3 KD flies [14]. SIN3 KD I and KD II refer to two established fly lines carrying distinct snapback constructs that target different regions of the *Sin3A* mRNA for degradation by RNAi [14]. SIN3 KD I females balanced on *CyO-Ras* were crossed to males carrying a deletion of the third chromosome balanced on *TM2-Ubx*, *TM3-Sb*, *TM3-Ser* or *TM6-Tb*. The cross yields progeny with four different genotypes (Figure 1). One of these genotypes yields flies that are *Sin3A* knockdown only and therefore will have curved wings as well as yellow eyes due to the presence of a single copy of the mini *white* gene in the *UAS-SIN3^RNAi^* and *Ser-GAL4* transgenes. These flies will also carry a third chromosome balancer and consequently will display the phenotype associated with the balancer. Another genotype yields flies that are *Sin3A* knockdown and heterozygous for a deletion on the third chromosome. These flies are identified by the absence of the *CyO-Ras* chromosome and third chromosome balancer phenotypes as well as the appearance of dark red eyes due to presence of three copies of the mini *white* gene; two from the SIN3 KD chromosome and one from the third chromosome having the deletion. If one or more of the genes within the deletion interact with *Sin3A* then this mutation could lead to modification of the *Sin3A* knockdown curved wing phenotype. In the other two genotypes resulting from the parental cross, the presence of the dominant *Ras* mutation leads to a rough eye phenotype in the flies carrying the *CyO-Ras* balancer. This allowed us to differentiate between flies that are curved due to loss of *Sin3A* as opposed to flies that are curly due to the presence of the balancer.

**Figure 5:**
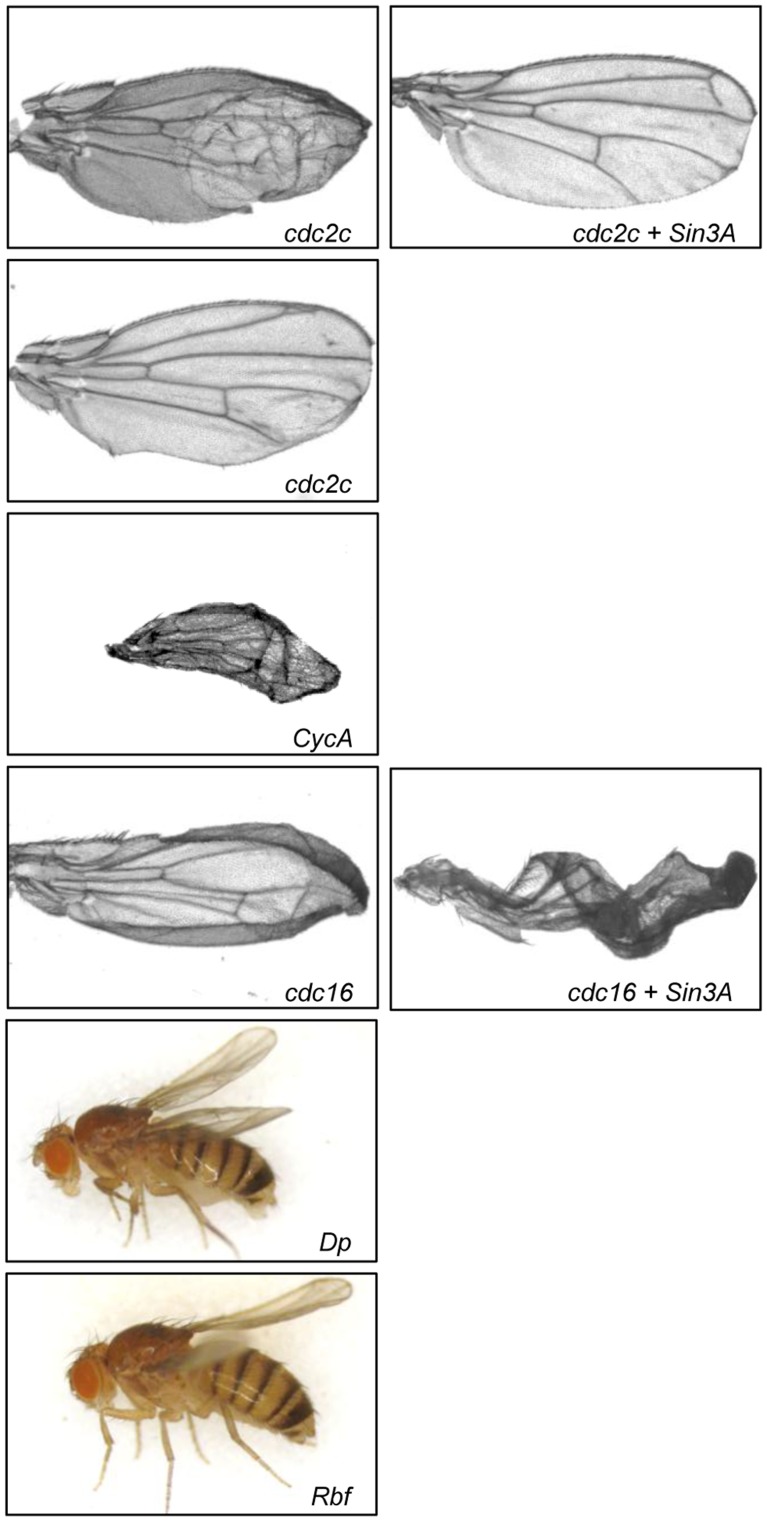
Wing development is sensitive to reduced expression of cell cycle regulators. Images of representative wings from progeny of *Ser-GAL4* X *UAS-RNAi* of the indicated gene (left panels). For each of these genes, the wing phenotype of the double knockdown was the same as for the single gene knockdown except where noted (right panels). For *cdc2c*, images representing the variable phenotypes in the population are shown.

### The Unbiased Screen Identified Regions on the Third Chromosome that Genetically Interact with *Sin3A*

In this first phase (phase I) of the screen we found 21 out of a total of 148 deletions tested that suppressed the penetrance of the curved wing phenotype to varying degrees (Table S2). Some of these represent overlapping regions on the third chromosome, narrowing the number down to 15 unique regions or cytogenetic intervals on the third chromosome that genetically interact with *Sin3A*. The interactions can be broadly categorized into two groups. For the first, partial suppression of the penetrance but not the expressivity (i.e. a milder or stronger curl in the wing) of the curved wing phenotype is observed. In this category any fly that showed suppression has completely straight wings. The second group includes flies with partial suppression of the expressivity of the curved wing phenotype but not the penetrance. Scoring suppression of the expressivity was difficult and we were unable to assign a scale for the extent of suppression. Thus, to accurately identify suppressors of the curved wing phenotype, we have taken into consideration only those regions that affect the penetrance of the phenotype and not those that modified its expressivity. A control cross was set up in which SIN3 KD I and II/*CyO-Ras* females were crossed to *GFP-RNAi* males, and the progeny carrying the SIN3 KD chromosome were scored for curved or straight wings. 90 to 94% of the progeny from this cross carrying the SIN3 KD (I and II respectively) chromosome had curved wings. To ensure that the genetic interaction observed was between *Sin3A* and a gene removed by the deletion, we performed additional controls in which we crossed the putative suppressors individually to the flies carrying transgenes used for *Sin3A* knockdown. The fly lines for the control crosses included *Ser-GAL4* and *UAS-SIN3^RNAi-I^*. *Ser-GAL4* is the wing imaginal disc driver line. *UAS-SIN3^RNAi-I^* flies carry the construct for expression of the inverted repeat to target *Sin3A* knockdown. The repeat is not expressed in the absence of GAL4. The progeny of these crosses were scored for any wing aberrations. In this initial phase of the screen, no wing phenotypes were observed in the progeny, suggesting that the assay identified true suppressors of the *Sin3A* knockdown phenotype.

In phase II of the screen we attempted to narrow down the cytogenetic intervals that interact with *Sin3A* by using smaller deletions within the regions identified in phase I (Table S2). Out of the 30 smaller deletions tested, 22 were able to suppress the curved wing phenotype. As with phase I, all deletion lines of phase II were subject to the same control crosses to ensure that the deletion alone did not lead to a wing phenotype.

Inspection of the deletions identified in phase II allowed us to generate a list of hundreds of genes that when reduced in expression could potentially modify the curved wing phenotype. To identify individual genes that interact with *Sin3A*, we selected a subset of the genes present in the deletions for analysis. We chose an individual gene based on the following criteria: (1) known or predicted biological function, for example, genes involved in regulating cell cycle, gene expression, wing development and signaling pathways based on information present in FlyBase [20], (2) availability of a characterized loss of function allele or an RNAi line for the gene and (3) presence within a particular deletion. We attempted to identify at least one interactor within each small deletion, so in some cases, we tested genes with unknown function. To this end, in phase III of the screen we have tested loss of function alleles and/or RNAi lines for a total of 81 genes for the ability to suppress the *Sin3A* knockdown curved wing phenotype (Table S2).

**Table 4:** Components of the Mediator kinase module genetically interact with *Sin3A*.

CG Number	Gene Symbol	Mediator Module	SIN3 KD I^a^		SIN3 KD II^a^	
			RNAi	LOF	RNAi	LOF
CG10572	*Cdk8*	Kinase	79±9^b^	n.t.	89±6^b^	n.t.
CG7281	*CycC*	Kinase	0^c^	n.t.	0^c^	n.t.
CG8491	*kto*	Kinase	0^c^	53±30	0^c^	34±16
CG9936	*skd*	Kinase	n.t.	82±5	n.t.	87±5
CG7957	*MED17*	Head	n.t.	0	n.t.	0
CG18267	*MED20*	Head	n.t.	2±2	n.t.	0
CG5057	*MED10*	Middle	n.t.	0	n.t.	0
CG7162	*MED1*	Middle-Tail Junction	n.t.	54±4	n.t.	49±3
CG3695	*MED23*	Tail	n.t.	0	n.t.	0
CG7999	*MED24*	Tail	n.t.	4±3	n.t.	3±1

a SIN3 KD I and II/*CyO-Ras* females were crossed to males carrying an RNAi or loss of function (LOF) allele for the indicated gene.

b The percentage of straight winged flies in the progeny of the cross that are knocked down for *Sin3A* and for the indicated gene is given. Results are an average of three trials. n>100. Standard deviation is indicated.

c Flies had a wing phenotype that was neither straight nor curved.

n.t., not tested.

We observed three classes of phenotypes in the double knockdown flies. These phenotypes included curved wings similar to the *Sin3A* knockdown phenotype, straight wings similar to wild type flies and wings with a completely distinct phenotype such as blistered or disrupted veins. For those crosses that yielded double mutant flies having straight wings or wings with a new phenotype, the control crosses with the individual mutant gene fly line were set up and scored. Importantly, all of the RNAi lines were tested with the same *Ser-GAL4* driver to determine if knockdown of the candidate *Sin3A* interacting gene in a wild type background resulted in a wing phenotype.

Results of phase III allow us to group the genes into four distinct categories. In the first are genes whose knockdown in the wing had no phenotype on their own but when combined with knockdown of *Sin3A* yielded some percentage of straight wings (Tables 1, S2). Genes of this first category are identified as suppressors of the *Sin3A* knockdown curved wing phenotype. The finding that reduction of these given genes suppressed the *Sin3A* curved wing phenotype is indicative of a genetic interaction between *Sin3A* and the tested gene. The second major category of genes includes all of the RNAi flies that yielded a new wing phenotype in the *Sin3A* knockdown background and exhibited a distinct phenotype when the gene was individually knocked down in the wild type background (Figure 2). The third category of genes in the group of double knockdown flies yielded flies with a new wing phenotype in the *Sin3A* knockdown background and they exhibited that same wing phenotype when the gene was knocked down in the wild type background. Given this result, we are unable to make any strong conclusions about the potential for interaction between *Sin3A* and these genes. The final category includes those genes that when mutated or reduced in expression did not suppress the *Sin3A* knockdown curved wing phenotype and so are not *Sin3A* genetic interactors (Table S2).

In summary, in phase III of our unbiased screen, we identified 38 genes that suppressed the *Sin3A* knockdown curved wing phenotype. Additionally, single reduction of 13 genes in wing imaginal discs resulted in an observable altered wing phenotype, indicating their importance in wing development. From this group of genes, 38% of the double knockdown flies yielded a phenotype that was distinct from either the *Sin3A* knockdown or the tested gene knockdown, suggesting that these genes might work in parallel pathways to regulate wing development. For some genes, the double knockdown resulted in full or partial lethality (Table S2), implying that Sin3A and the protein encoded by the second gene work in parallel pathways required for viability.

Genes that showed a suppression of the *Sin3A* knockdown curved wing phenotype fell into a number of distinct functional categories (Table 1). Only those genes which when reduced in expression resulted in greater than 10% suppression of both SIN3 KD I and KD II are included on this list. Based on RNA-sequencing data provided by the modEncode consortium and given on FlyBase, the large majority of suppressors are expressed at moderate levels or higher in samples isolated from pooled imaginal discs [20], [21]. Only three genes, *CG32024*, *CG10233*, and *CG11905*, had no detectable level of RNA in imaginal disc cells. Possibly they act at a prior stage of development, allowing their decrease to suppress the *Sin3A* knockdown phenotype. Three other genes, *mRpS9*, *mRpL19* and *ALiX*, were previously identified as targets of Sin3A [22]. These genes exhibited increased expression in RNAi knockdown S2 cells compared to wild type controls. In this current study utilizing the *Sin3A* wing imaginal disc knockdown system, perhaps the expression of the gene is restored to the level in wild type discs, thus suppressing the *Sin3A* knockdown curved wing phenotype. As predicted, *Sin3A* interacted with genes involved in regulation of transcription, cell signaling, cell division and proliferation. Additional categories represented included GTP regulation, apoptosis, DNA repair, translation, larval development, metabolism, proteolysis and chitin biology.

**Figure 6:**
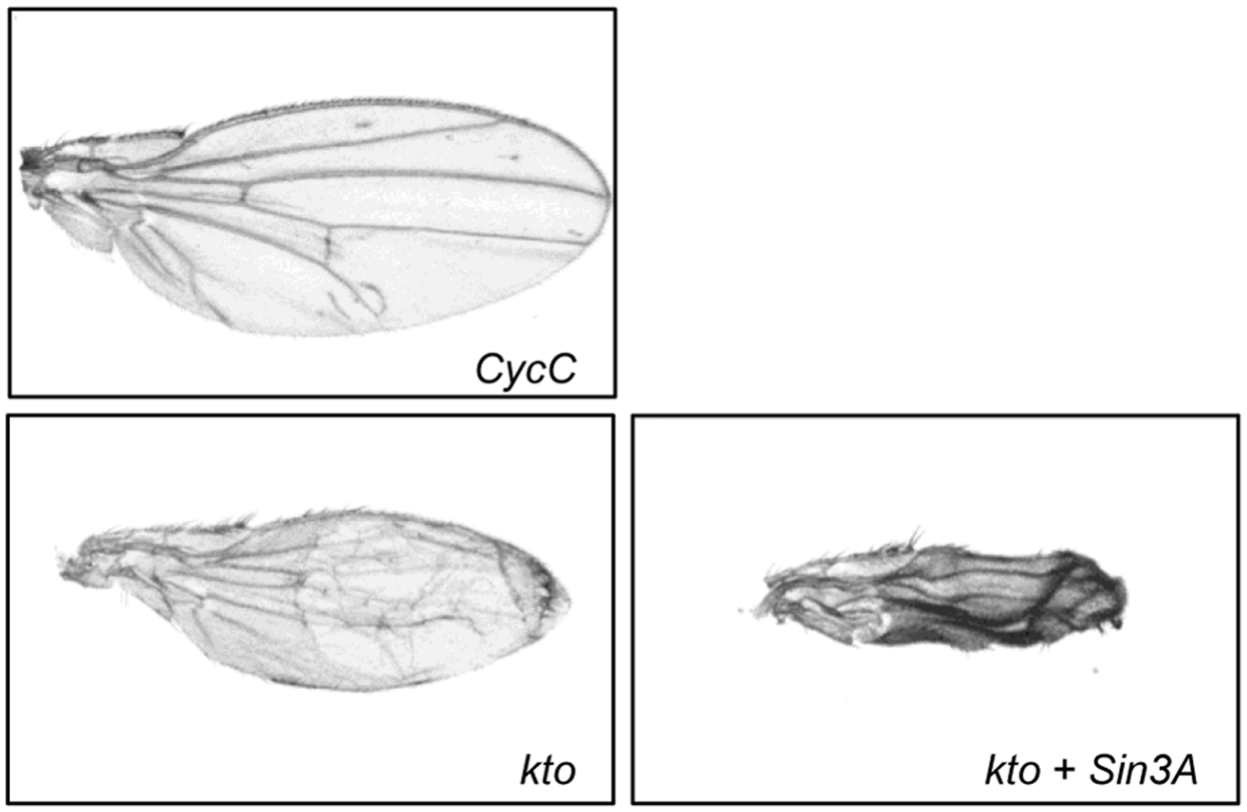
Two components of the Mediator accessory kinase module are important for wing morphology. Images of representative wings from progeny of *Ser-GAL4* X *UAS-RNAi* of the indicated gene. For *CycC*, the wing phenotype of the double knockdown was the same as for the single gene knockdown. The phenotype of the single and double knockdown phenotype with *kto* is shown.

**Figure 7:**
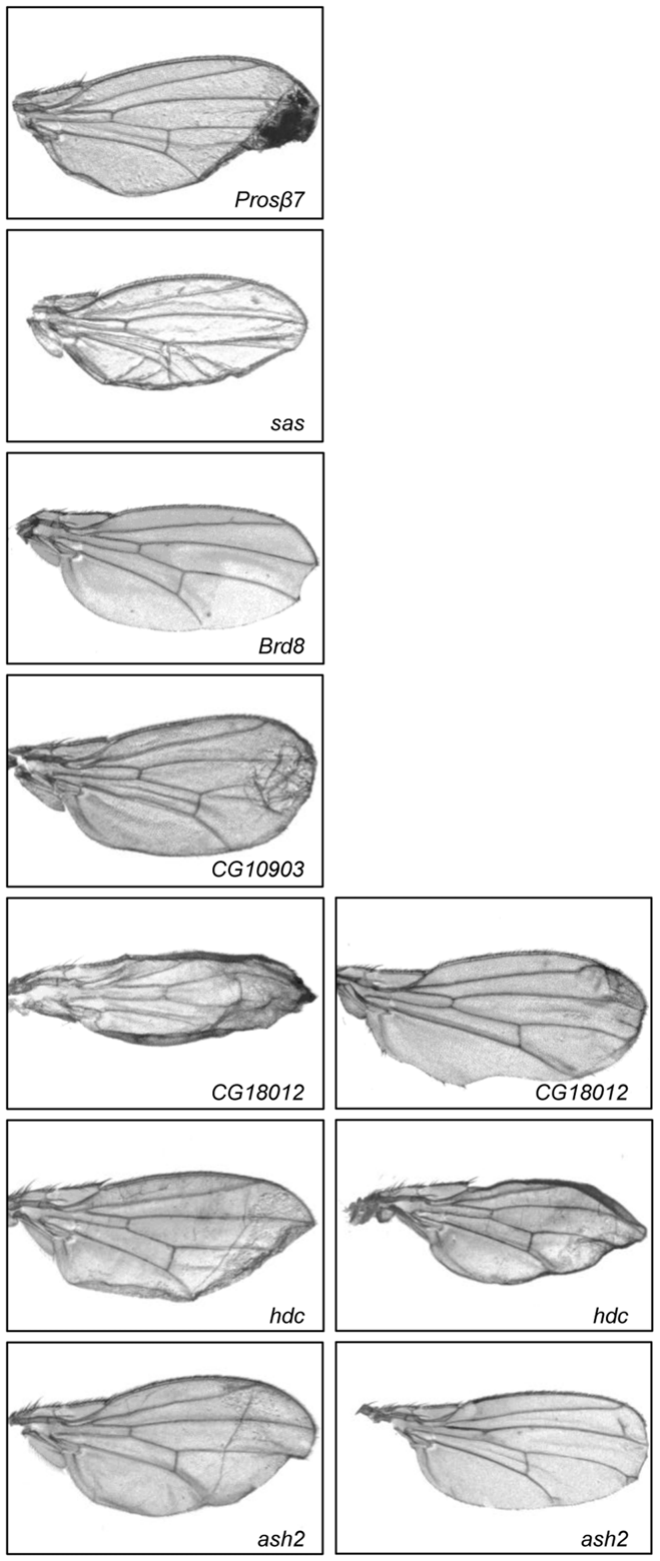
Multiple genes that reside along the third chromosome are required for normal wing morphology. Images of wings from progeny of *Ser-GAL4* X *UAS-RNAi* of the indicated gene. For each of these genes, the wing phenotype of the double knockdown was the same as for the single gene knockdown. In cases where the phenotype was variable in the population, multiple representative images are shown.

### *Sin3A* Genetically Interacts with Other Genes Involved in the Process of Transcription

As expected, mutant alleles in number of genes that function in a variety of processes involved in transcription and regulation of gene expression were found to suppress the curved wing phenotype. These processes included gene specific regulation by DNA binding factors (*nerfin-2, PSEA-binding protein 95 kD (Pbp95), eyegone (eyg), twin of eyg (toe)*), transcription itself (*meiosis I arrest* (*mia)* and *Rpb4*), RNA interference (*FBX011 ortholog* (*FBX011*)), chromatin remodeling (*osa*) and histone modification, such as histone acetylation (*Pcaf and CG3909*) and histone methylation. Interestingly, multiple genes (*Arginine methyltransferase 4 (Art4), Alhambra* (*Alh) and Lysine (K)-specific demethylase 2 (Kdm2*)) involved in methylation, including both methyltransferases and demethylases, were identified.

The DNA binding factors that suppress the *Sin3A* knockdown curved wing phenotype might act to affect transcription of genes important for limiting cell proliferation as the wing imaginal disc cells proceed through the larval stage. *Nerfin-2* is a little studied gene predicted to encode a transcription factor involved in neural development based on the presence of a zinc finger domain and expression in a limited number of brain neurons [23]. *Pbp95* encodes a DNA-binding transcription factor that is part of a protein complex required for expression of *U1* and *U6* snRNAs important for the spliceosome [24]. *eyg* and *toe* are two similar genes that encode homeodomain containing transcription factors [25]. As their name suggests, these factors have been shown to be very important for eye development [25], [26]. While expression of *eyg* and *toe* have been found to be quite high in the larval eye disc, expression can be detected in the wing disc and misexpression of *eyg* and *toe* has been found to lead to abnormal thorax development [25], [27].

MIA is one isoform of TAF6, a component found in TFIID, the general transcription factor [28]. While phenotypes associated with mutations in *mia* are linked to spermatogenesis, the transcript is found in other larval tissue in addition to the prominent expression in the male germ line [29], [30]. Of note, like Sin3A, MIA is required for G2/M progression [29]. Why mutation in one factor involved in G2/M progression would suppress a phenotype associated with another factor involved in the same process is an interesting question. RPB4 is a subunit of RNA polymerase II, important for transcription [31], [32]. FBX011, a predicted ubiquitin ligase, was shown to have putative role in transcription regulation as it is important for RNAi silencing of gene expression by siRNA and miRNAs [33].

In addition to histone modification, a second major enzymatic activity to affect chromatin and regulate transcription is that which is carried out by the ATP-dependent nucleosome remodeling complexes. It has long been appreciated that histone modifiers and remodeling complexes work in concert to affect transcriptional outcomes [34]. We identified Osa, which encodes a component of a subset of *Drosophila* Brahma complexes [35] and the *Drosophila* version of SWI/SNF, as a suppressor of the curved wing phenotype. Osa has been found to bind to AT-rich DNA sequences and to be important for cell growth and survival in developing wing imaginal discs [35], [36]. It is interesting that one gene important for cell survival acts as suppressor of a second gene important for this same critical function.

The *Pcaf* gene was present in one of the large DrosDel deletions. *Pcaf* encodes a histone acetyltransferase [37]. Previously, we determined that *Pcaf* haplo-insufficiency could suppress the curved wing phenotype due to SIN3 knockdown [14]. The finding that alleles of *Pcaf* suppress the phenotype due to *Sin3A* knockdown strongly suggests that KAT and Sin3A HDAC complexes act in opposition. Complexes containing Pcaf and Sin3A have been found to target similar histone amino acid residues. The Pcaf containing dSAGA complex targets lysine 9 and 14 of histone H3 [38]. The same residues are targets of the Sin3A HDAC complex [39]. *CG3909* is a second gene with a link to SAGA and histone acetylation. This factor was identified in an analysis of proteins found to interact with the SAGA complex subunit, Ada2b, immunopurified from muscle or neuronal cells [40]. We tested an *Ada2b* loss of function allele but did not observe a genetic interaction with *Sin3A* in the wing (Table S2). *CG3909* expression was reduced using an RNAi construct, so the amount was possibly reduced more than that of *Ada2b*. Little is known about *CG3909* and how it might function in transcription. Although it has been shown to be important for normal growth of wing disc cells [41], we did not observe a wing phenotype when expression of *CG3909* was reduced using the *Ser-GAL4* driver in the wild type background.

Interestingly, multiple genes, including *Art4, Alh and Kdm2*, involved in histone methylation were found to suppress the *Sin3A* knockdown curved wing phenotype. ART4 is an arginine methyltransferase that has been shown to be important for expression of ecdysone receptor (EcR) regulated genes during development [42], [43]. In this way, ART4 is similar to Pcaf in that it acts in opposition to Sin3A, which has been shown to bind EcR targets for repression [5], [6]. ALH was initially isolated as a zinc finger containing protein required for neuronal even-skipped expression, indicating a role for this factor in transcription [44]. Subsequent work found this factor to be a component of the H3K79 methyltransferase complex DotCom [45]. H3K79 trimethylation is linked to gene activation [46]. Accordingly, a reduction in a component of this complex may act to counteract the upregulation of gene expression following *Sin3A* knockdown.

KDM2 is a histone demethylase that targets histone H3K36 dimethylation in S2 cells and H3K4 trimethylation in *Drosophila* larvae and adults [47], [48]. *Kdm2* has been found to genetically interact with another H3K4me3 demethylase, *little imaginal discs* (*lid*) [49]. The viability of flies carrying mutations in both *Kdm2* and *lid* is less than that of the single mutants and the lethality cannot be rescued by introduction of a *lid* allele carrying a mutation in the demethylase domain. This finding suggests that *Kdm2* and *lid* are partially redundant in function with respect to H3K4me3 demethylase activity. LID has been also isolated as a component of the SIN3 220 complex [39]. Perhaps surprising given the suppression of the *Sin3A* knockdown phenotype by knockdown of *Kdm2*, our laboratory has determined that, like *Sin3A*, reduction of *lid* in wing imaginal disc cells produces a curved wing phenotype in the adult (unpublished data). It is possible that KDM2 and LID have some gene specific functions with respect to wing development, with LID acting in concert with Sin3A and KDM2 acting in opposition. While we do not fully understand the reasons as to why these two demethylases have different roles with respect to Sin3A and wing development, these findings underscore the important link between methylation and acetylation.

### *Sin3A* Genetically Interacts with Genes Involved in Wing Development

In addition to the above genes whose mutation suppresses the *Sin3A* knockdown curved wing phenotype but do not affect wing development on their own, we identified a few genes encoding proteins involved in transcription that affect wing development when singly reduced in expression and also result in an distinct phenotype when the mutation is combined with *Sin3A* knockdown. Two factors involved in basal transcription fall into this category (Table S2 and Figure 2). TFIIA-L is a general transcription factor and RPB10 is a subunit of RNA polymerase II [28], [50]. Interestingly, the combination of *Sin3A* and *Rpb10* knockdown results in partial lethality. While we are using the *Ser-GAL4* driver to specifically induce the RNAi pathway in the wing, *serrate* expression has been demonstrated in other tissues [30], [51], likely leading to the observed synthetic lethality. The few survivors of the *Sin3A*, *Rpb10* double knockdown have a wing phenotype that is more severe than either of the single gene knockdowns (Figure 2). As for the wing phenotypes observed in the single gene knockdowns, neither *TfIIA-L* nor *Rpb10* has previously been found to have a role in wing development.

Through this screen three additional genes were identified as having a role in wing development and affecting the *Sin3A* knockdown phenotype (Figure 2). *Ferritin 1 heavy chain homologue (Fer1HCH)* is an essential gene involved in iron homeostasis [52]. This gene was previously found to be a target of Sin3A as expression increased in *Sin3A* knockdown cultured cells [22]. Mitochondrial transcription factor B2 (MTTFB2) is important for transcription of genes from the mitochondrial genome [53]. The finding that the combination of reduction of *mtTFB2* and *Sin3A* results in synthetic lethality is interesting in light of the previous finding linking regulation by Sin3A to mitochondrial function [22], [54]. *DNA polymerase α 73kD* (*DNApol-α73*) also shows synthetic lethality with *Sin3A*. Based on sequence similarity to yeast and mammalian genes, this factor is predicted to play a role in DNA replication [55]. FER1HCH, MTTFB2 and DNAPOL-α73 have not previously been implicated in wing development. Taken together, the results indicate that as predicted, multiple complex processes are important for wing development. They also suggest that Sin3A likely functions to regulate the expression of genes involved in multiple steps along the developmental path to a normal wing.

### *Sin3A* Genetically Interacts with Genes Involved in Multiple Signaling Pathways

*Sin3A* was found to interact with genes involved in distinct signaling pathways including *pelle (pII), Band4.1 inhibitor LRP interactor (Bili), 5PtaseI, ALG-2 interacting protein X (ALiX)* and *CG7910*. PLL is a kinase involved in the Toll signaling pathway [56]. The Toll pathway has been implicated in the *Drosophila* immune response to Gram-positive bacterial or fungal infection [57], [58]. Triggering this pathway results in the upregulation of genes via the nuclear factor κB–related protein, Dorsal-related immune factor [57]. 5PTASEI is involved in inositol signaling [59]. Interestingly, RNAi knockdown of *Sin3A* in S2 cells affected another gene involved in this pathway, *inositol-3-phosphate synthase* [22]. BILI is a negative regulator of the Wnt signaling pathway, critical for wing development, as discussed below [60]. ALIX has been implicated in JNK signaling [61]. *Sin3A* has been linked to JNK signaling as a positive hit in a genome-wide RNAi screen in *Drosophila* S2 cells [12]. Additionally, the enzymatic component of the Sin3A complex, Rpd3, has been found to directly affect this pathway [62]. As mentioned earlier, *ALiX* is one of three of the suppressor genes that was previously found to be a target of Sin3A in the S2 expression profiling analysis [22]. Little is known about CG7910 but based on sequence it is predicted to have fatty acid amide hydrolase activity. In humans, enzymes with this activity have been shown to be important for lipid signaling [63]. For these pathways, it is possible that Sin3A typically represses the targets in the absence of the activating signal. The reduction of the identified signaling component may dampen an inappropriately activated response due to *Sin3A* knockdown.

The Wnt pathway has been implicated in cell division in the wing disc and development of the wing [64]. Briefly, binding of the wingless (Wg) ligand to the Frizzled/low density lipoprotein (LDL) receptor-related protein (LRP) inhibits the degradation of Armadillo (ARM) resulting in its accumulation in the cytoplasm and nucleus. Nuclear ARM interacts with TCF to influence transcription of Wnt responsive genes [65]. BILI, encoded by a gene that suppressed the curved wing phenotype (Table S2), acts as a negative regulator of the Wnt pathway by destabilizing the interaction between Wg and LRP5/6 such that the downstream signals cannot be turned on [60]. As there is a paucity of existing evidence demonstrating that the Sin3A complex plays a role in Wnt signaling, we decided to determine if additional genes that encode negative regulators of the Wnt pathway genetically interact with *Sin3A*. We tested if reduced expression of known Wnt negative regulators by RNAi or a loss of function allele could modify the *Sin3A* knockdown curved wing phenotype.

Using the QueryBuilder tool on FlyBase [20], we generated a list of genes that act as negative regulators of Wnt signaling (Table 2). Four of the ten additional factors tested were able to suppress the *Sin3A* knockdown curved wing phenotype to a level above 10% and therefore are classified as *Sin3A* interactors. Two of the four, *pangolin (pan,* which encodes TCF*) and Axin (Axn),* have been shown to encode proteins important for regulation of Wg expression and thus important for the growth and cell-fate specification of the wing [66], [67]. While loss of APC2 alone results in no observable phenotype in larval imaginal wing discs [68], [69], loss of APC2 in combination with APC1 in the developing wing disc results in deregulation and consequential accumulation of ARM to activate Wg signaling [70]. Reduction of *RanBP3* by RNAi in *Drosophila* embryos results in naked cuticle phenotype and a broader *engrailed* expression domain, both caused by activation of Wnt signaling [71].

When expression of two of the negative Wnt regulators, *tumbleweed (tum) and shaggy (sgg)*, was reduced in the *Sin3A* knockdown background, the flies exhibited an abnormal wing phenotype distinct from the curved wing (Figure 3). When tested individually with the *Ser-GAL4* driver, the resulting phenotypes were essentially the same as the double knockdown. *tum* has been shown to have a putative role in wing disc regeneration based on expression profile analyses performed in a regenerative wing model system [72]. Overexpression of *sgg* has been shown to cause failure of the epithelial layer to separate from the cuticle which prevents the dorsal and ventral cuticle surfaces from bonding [73].

Knockdown of *fat (ft)* alone resulted in a curved wing that is held out horizontally (Figure 3). The double mutant did not exhibit the held out wing, rather the wing was curved similar to the *Sin3A* knockdown phenotype. *Ft* has been found to play a role in wing patterning [74]. Taken together, these results indicate that Wnt genes encode factors that are not only involved in regulation of wing development, but also that this regulation may be due in part to interactions with the Sin3A epigenetic regulatory factor.

Negative regulators of the Wnt pathway interfere with signal transduction at various stages of the pathway ultimately resulting in downregulation of Wnt responsive genes [65]. Thus, loss of negative regulators of the Wnt pathway results in an upregulation in Wnt responsive genes. One hypothesis to explain the interaction between *Sin3A* and negative Wnt regulators is that knockdown of *Sin3A* results in the downregulation of one or more Wnt target genes or components of the pathway itself, so that when a negative regulator of Wnt is mutated, upregulation of the Wnt pathway occurs and the wings are restored to normal. To test this hypothesis, we assayed the gene expression of some Wnt pathway components, including effectors and targets, by qRT-PCR in control and *Sin3A* knockdown wing discs. *pan* and *nemo*, which encode both effectors and targets of the pathway, *Bili*, an effector, and targets *stripe* (*sr*) and *diminutive* (*dm*) were downregulated upon reduction of *Sin3A* whereas the other tested genes were unaffected (Figure 4). These results are consistent with our hypothesis, but suggest a novel role for Sin3A in gene activation in *Drosophila* whereby recruitment of Sin3A to Wnt targets results in upregulation. A role for Sin3 in transcription activation in other model systems has been recently demonstrated. Mammalian Sin3 proteins are recruited to promoters and required for activation of pluripotency genes in embryonic stem cells and at number of muscle specific genes in the muscle lineage during differentiation [75], [76]. As for the Wnt targets, an alternate explanation is that Sin3A may function further upstream of the Wnt pathway such that loss of Sin3A prevents activation of the pathway.

### Genes Involved in the Cell Cycle Genetically Interact with *Sin3A*

Previous work on *Drosophila* Sin3A suggests it plays an important role in regulating the cell cycle in cultured cells and the developing wing disc [13], [14]. *Sin3A* genetically interacts with *stg* and *cdc2*, in that over expression of either of these factors important for the G2 to M transition suppressed the *Sin3A* knockdown curved wing phenotype, albeit not completely [14]. From the screen, we uncovered *Sin3A* interacting genes encoding factors that regulate other phases of the cell cycle and cell division. These genes include *humpty dumpty (hd), Sas-4, abnormal spindle (asp)* and *Cyclin B3 (CycB3)*. *hd* expression peaks during the G1 and S phase of the cell cycle [77]. As it was identified in a screen isolating factors involved in DNA amplification, HD is believed to be critical for cell proliferation [77]. Sas-4, ASP and CYCB3 are known to be important for mitosis and have been specifically linked to mitotic spindle organization [78], [79], [80], [81]. Data from the unbiased screen combined with our previously published results indicate that Sin3A is likely involved either directly or indirectly with regulation of multiple phases of the cell cycle.

To extend the analysis of whether *Sin3A* interacts with genes involved in other phases of the cell cycle, we performed a targeted screen to test if mutations in known cyclins and/or cyclin dependent kinases (CDKs) and other key cell cycle regulators could modify the curved wing phenotype. The candidates tested, the position in the cell cycle in which they act and the wing phenotypes of the progeny in combination with *Sin3A* knockdown are given in Table 3 and Figure 5. Consistent with our hypothesis, in addition to the G2/M phase regulators, regulators of G1/S and M phase also interacted with *Sin3A*. Of the eight additional genes tested, four were found to suppress the *Sin3A* knockdown curved wing phenotype (Table 3). Two of these suppressors, *CycE* and *CycJ,* have not been previously identified as being important for wing development. On the other hand, *CycB* and *cdc16* have been uncovered in genetic screens as potential effectors of wing morphogenesis and differentiation [82], [83].

Reduction by RNAi of three genes *(cdc2c, Cyclin A (CycA) and cdc16)* in the *Sin3A* knockdown background resulted in a severe wing phenotype (Table 3, Figure 5). Not surprisingly, as individual knockdown of each of these genes also yields a wing phenotype, these genes have been previously identified in genetic screens as important for wing development and differentiation [82], [83]. *cdc16*, which encodes anaphase promoting complex/cyclosome (APC/C), important for mitosis [84], is an interesting gene in that the loss of function allele suppressed the *Sin3A* knockdown curved wing phenotype while the RNAi allele resulted in a severely disrupted wing (Table 3, Figure 5). These data suggest that the dose of *cdc16* is very important for its function. For *cdc16* as well as *cdc2c* the double knockdown resulted in an observable phenotype distinct from that of the single knockdowns (Figure 5).

Finally, while knockdown of *DP transcription factor (Dp)* or *Retinoblastoma-family protein (Rbf)* did not alter the *Sin3A* knockdown curved wing phenotype, single reduction of either of these factors resulted in a curved wing phenotype (Figure 5). Both have previously been linked to wing development [17], [85], [86]. The finding that reduced expression of DP, RBF or Sin3A produces a similar phenotype indicates that these factors may work in a similar pathway for wing development. This idea is consistent with previously published results from studies on mammalian Sin3 proteins. mSin3B was found to interact with pRB family members and to be recruited to E2F regulated genes [87], [88]. These findings underscore the evolutionarily conserved function for the Sin3 complex in the regulation of cell proliferation.

Taken together, these results suggest the following. First, all of these cell cycle genes are important for some process of wing development. Wing phenotypes result from reduced expression of the gene, or the reduced expression suppresses the wing phenotype brought about by knockdown of *Sin3A*. Second, because all of these factors have some effect, these results suggest that wing development is highly sensitive to alterations in the cell cycle/proliferation program. Third, the observed genetic interactions support the previously published findings indicating a connection between Sin3A and cell cycle control. Finally, as genes encoding regulators of multiple phases of the cell cycle were found to interact with *Sin3A*, histone deacetylation likely plays a role at distinct transition points in the cell cycle.

### *Sin3A* Genetically Interacts with Components of the Mediator CDK8 Accessory Module

Multiple CDKs are expressed in a cell, some of which are directly involved in regulating the cell cycle while others may have an indirect role. Some have also been implicated in regulating transcription. One such CDK is *Cdk8*, which shares 32% homology with *cdc2* [89]. Since *Sin3A* genetically interacts with *cdc2*, we tested if it could also interact with *Cdk8*. We determined that the curved wing phenotype is suppressed by RNAi mediated downregulation of *Cdk8* (Table 4). CDK8 is a member of the *Drosophila* transcription Mediator complex [90]. It associates with three other factors including Cyclin C (CYCC), Kohtalo (KTO or MED12) and Skuld (SKD or MED13) to form the “kinase” module of Mediator [91]. Similar to knockdown of *Cdk8*, mutations in two of the other members of the kinase module, *kto* and *skd*, were also able to suppress the curved wing phenotype (Table 4). Of note, single RNAi knockdown of either *CycC* or *kto* in the wing disc using the *Ser-GAL4* driver resulted in an altered wing phenotype (Figure 6). Similar to *cdc16* discussed above, haplo-insufficiency of *kto* suppressed the *Sin3A* knockdown phenotype while RNAi knockdown of *kto* alone resulted in an abnormal wing. The results suggest that dose of *kto* is important for its function. The findings also indicate that like Sin3A, CYCC and KTO are required for normal wing development. *kto* and *skd* were previously identified as effectors of wing development in dorsal-ventral boundary formation [92]. The interaction data further suggest that multiple members of the kinase module genetically interact with *Sin3A* during wing development.

While Mediator is essential for the majority of RNA polymerase II dependent transcription, individual genes depend on RNA polymerase II association with distinct Mediator modules and subunits [91]. The CDK8 module is essential for the activation of Wnt target genes in the wing discs [93]. It is also essential for the development of external sensory organs on the notum, which arise from the wing discs [94]. The investigators of that study found that, unlike the CDK8 and CYCC components of the Mediator kinase module, SKD and KTO are not required for cell proliferation or survival, rather they regulate the formation of boundaries in the eye disc. These results suggest that even within a single Mediator module, there are distinct roles for individual subunits. Taken together, the published data along with our findings of a genetic interaction with *Sin3A* indicate that the Mediator kinase module is important for regulation of gene transcription, and that this module interfaces with the Sin3A epigenetic regulator as part of this function.

One possible explanation for the interaction of *Sin3A* with these genes of the CDK8 Mediator module is that loss of Sin3A results in the upregulation of genes that are normally activated by the Mediator accessory complex. A mutation in a gene encoding a CDK8 module component in a *Sin3A* knockdown background may restore the expression of these genes to near normal levels, resulting in the suppression of the curved wing phenotype. The genetic interaction between *Sin3A* and *kto*, *skd* and *Cdk8* suggests that the effect of loss of Sin3A on the cell cycle is not due solely to defects in cell proliferation but also due to its role in regulating a specific set of genes involved in the process.

We also tested other Mediator components for their ability to interact with *Sin3A*. We selected subunits that represented the tail, head and middle modules of Mediator [91]. Interestingly, while genes encoding three of four of the CDK8 module factors interacted with *Sin3A*, only one of the other tested mediator subunits, *MED1*, was found to suppress the *Sin3A* knockdown curved wing phenotype (Table 4). MED1 (TRAP220) functions as a global coactivator of the superfamily of nuclear hormone receptors [95]. It acts after the ligand-independent binding of nuclear receptors and coprepressors, like Sin3A, to target DNA sites. This is followed by the ligand-mediated dissociation of the corepressors accompanied by binding of coactivator such as KATs. This activation culminates with the binding of MED1 and other TRAPs that facilitate preinitiation complex formation or function.

### Identification of Genes Involved in Wing Development

Seven RNAi fly stocks yielded an abnormal wing phenotype in the *Sin3A* knockdown background and exhibited that same wing phenotype when the gene was knocked down in the wild type background (Figure 7). Because the phenotype was the same in the single and double mutant, we are unable to conclude that these factors genetically interact with *Sin3A*. Of note, however, is that three of the seven genes have not been previously shown to affect wing development. This analysis has thus uncovered novel genes that play a role in the process of wing growth and differentiation.

Knockdown of *Proteasome β7 subunit* (*Prosβ7*), one of the fourteen 20S proteasome subunit genes, caused lethality and in survivors, crumpled blackened wings (Figure 7). Because the proteasomal degradation pathway is essential for many cellular processes including the cell cycle, it is perhaps not surprising that we observed this phenotype [96], [97]. S*tranded at second (sas)* is another novel effector of wing morphogenesis. It encodes a cell surface protein that functions as a receptor [98]. Three major *sas* transcripts are expressed in stage- and tissue-specific patterns throughout development in cuticle secreting epidermal tissues [98]. SAS likely functions to enable the complete separation of the layers of the epithelia and cuticle during wing maturation. The third novel gene, *Brd8,* was identified in a genome-wide RNAi screen in cultured *Drosophila* cells as a gene required for repression of E2F activity [99]. The E2F family of transcriptional regulators plays a crucial role in cell proliferation and differentiation and BRD8 is likely affecting wing development through defects in these pathways [17].

The other four genes that had an altered wing phenotype that was the same in the single knockdown and when combined with reduced *Sin3A* expression, all have some previously identified connection to the process of wing growth and differentiation. *CG10903* and *CG18012* have been linked to wing disc regeneration in a temporal regeneration model [72]. Based on sequence similarity, CG10903 has putative S-adenosylmethionine-dependent methyltransferase activity. It has also been identified in a *Drosophila* neuroblast RNAi screen as being important for cell proliferation [100]. Additionally, this gene was previously found to be a target of Sin3A as expression increased in *Sin3A* knockdown cultured cells [22]. Based on sequence similarity, CG18012 is predicted to have beta-1,4-mannosyltransferase activity and to affect protein glycosylation. *Headcase* (*hdc*), was previously found in a p-element insertion screen as causing general deformation in the wing shape, albeit only in males [101]. In our screen, the wing phenotype observed with *hdc* knockdown was equivalent in both sexes.

The role of the fourth gene, *absent, small, or homeotic discs 2 (ash2)*, in wing development is well established. *ash2* is another gene that we have identified where the loss of function allele suppressed the *Sin3A* knockdown curved wing phenotype while the RNAi allele resulted in a disrupted wing (Table S2 and Figure 7). These data suggest that the dose of *ash2* is essential for its function. *ash2* was first identified in a screen for factors involved in imaginal disc development and has subsequently been found to be required for wild type wing morphology [102], [103]. Additionally, a connection between ASH2 and Sin3A was made by the discovery of a large amount of overlap in the gene expression profiles of targets of these factors [104]. As ASH2 is a component of a histone methyltransferase complex [45], the genetic interaction between the genes encoding the two factors is further support for the link between histone acetylation and methylation.

A final gene that falls into the category of a fly yielding the same phenotype when mutated individually or when in combination with *Sin3A* is *RNA polymerase II 18kD subunit (RpII18)*, which encodes a subunit of RNA polymerase II [105], [106]. *RpII18* has been identified as a likely effector of wing shape based on microarray expression analysis of late third instar wing discs [107]. We did not observe a wing phenotype in our experiments, however, as *RpII18* knockdown with the *Ser-GAL4* driver, either individually or in combination with *Sin3A*, resulted in lethality (Table S2).

### Conclusions

Sin3A has been implicated in development in various organisms including *Drosophila* [2], [3], *Xenopus* [108], chick [109] and mammals [110], [111], [112]. Data from many of these previous studies implicate Sin3A in regulating various aspects of the cell cycle that affect development. Evidence exists that Sin3A is critical during development by functioning in signaling pathways possibly by regulating specific developmental genes. In this study, we set out to identify novel genes of Sin3A regulation and signaling pathways in which Sin3A may function. We have found that *Sin3A* genetically interacts with genes involved in development including histone modifying enzymes, signaling pathway effectors, cell cycle regulators and components of the transcription machinery. This finding suggests that Sin3A plays a wide variety of roles in a developing tissue such as the wing disc and that its function is not limited to regulating the cell cycle. Loss of Sin3A results in misregulation of Wnt responsive genes suggesting that Sin3A plays a role in the Wnt pathway in the developing wing disc. Genes involved in regulating multiple stages of the cell cycle are able to suppress the curved wing phenotype suggesting that Sin3A not only regulates G2/M progression as has been previously reported, but also other phases of the cell cycle including G1/S and mitosis. The genetic interaction between *Sin3A* and genes encoding components of the Mediator accessory kinase module suggests a role for Sin3A in counteracting the activation of genes by this complex. Further analyses of these interactions will shed light on the role of Sin3A in *Drosophila* development.

## Supporting Information

Table S1 **Primers used for Wnt gene expression analysis.**
(DOCX)

Table S2 **Deficiencies and alleles that result in suppression of the** ***Sin3A*** **knockdown curved wing phenotype.**
(XLSX)
