# Supplementary material for: Identification of Genetic Suppressors of the Sin3A Knockdown Wing Phenotype
Source: PLoS One. 2012 Nov 15;7(11):e49563. doi: 10.1371/journal.pone.0049563 (PMC3499482; doi:10.1371/journal.pone.0049563)
Supplement: Table S1 — Primers used for Wnt gene expression analysis. (DOCX) [file pone.0049563.s001.docx]

**Table S1: Primers used for Wnt gene expression analysis.**

| **Gene** | **Primer name** | **Primer sequence*^a^*** |
| --- | --- | --- |
| *arm* | Forward | AGTTCACACGGAGGTCGC |
|  | Reverse | CCACTGGGCTGCTGATCT |
| *Bili* | Forward | GGGAACACTGCAGTATAATCG |
|  | Reverse | GCGACACTTCACATCCGT |
| *dm* | Forward | GCGCCCTACAGTTCCAGA |
|  | Reverse | TTGGCCACCGATTTCACT |
| *en* | Forward | ATCCACCACCACAGAGGG |
|  | Reverse | GTGGACGCTTCTCGTCGT |
| *nmo* | Forward | CTGACATCCGTGCAGCAG |
|  | Reverse | GTGGATTGATGCACAGCG |
| *ndk* | Forward | CAAGATGTTGCGAAGGGC |
|  | Reverse | CGAGGCAGTGGTCCTGGT |
| *ovo* | Forward | AGCAAAGTCTTGCAGCGG |
|  | Reverse | GGCCAGCGGGTTCTTAAT |
| *sr* | Forward | GAGCCACCGCCCATTACT |
|  | Reverse | CCTGGGGTTCCAAAGACA |
| *Taf1* | Forward | GTGGAGGAGCCAAGGGAGCC |
|  | Reverse | TCCCGCTCCTTGTGCGAATG |
| *pan* | Forward | CCGCAAATGGGTATAGCG |
|  | Reverse | TGTCACAATGCTGATCCGTT |
| *Ubx* | Forward | CATTCTACCCCTGGATGG |
|  | Reverse | ATGCCGCCGTATTGTGTT |
| *wg* | Forward | GTCAGGGACGCAAGCATAAT |
|  | Reverse | GCGAAGGCTCCAGATAGACA |

*^a^* Forward and reverse primers (listed 5’ to 3’) were designed using Primer3 software (http://workbench.sdsc.edu/)
